# Sex Differential Genetic Effect of Chromosome 9p21 on Subclinical Atherosclerosis

**DOI:** 10.1371/journal.pone.0015124

**Published:** 2010-11-30

**Authors:** Hsiu-Fen Lin, Pei-Chien Tsai, Ruey-Tay Lin, Gim-Thean Khor, Sheng-Hsiung Sheu, Suh-Hang Hank Juo

**Affiliations:** 1 Department of Neurology, Kaohsiung Medical University Hospital, Kaohsiung, Taiwan Authority; 2 Department of Neurology, College of Medicine, Kaohsiung Medical University, Kaohsiung, Taiwan Authority; 3 Department of Medical Research, Kaohsiung Medical University Hospital, Kaohsiung, Taiwan Authority; 4 Division of Cardiology, Department of Internal Medicine, Kaohsiung Medical University Hospital, Kaohsiung, Taiwan Authority; 5 Department of Internal Medicine, College of Medicine, Kaohsiung Medical University, Kaohsiung, Taiwan Authority; 6 Department of Medical Genetics, College of Medicine, Kaohsiung Medical University, Kaohsiung, Taiwan Authority; Istituto Dermopatico dell'Immacolata, Italy

## Abstract

**Background:**

Chromosome 9p21 has recently been shown to be a risk region for a broad range of vascular diseases. Since carotid intima-media thickness (IMT) and plaque are independent predictors for vascular diseases, the association between 9p21 and these two phenotypes was investigated.

**Methodology/Principal Findings:**

Carotid segment-specific IMT and plaques were obtained in 1083 stroke- and myocardial infarction-free volunteers. We tested the genotypes and haplotypes of key single nucleotide polymorphisms (SNPs) on chromosome 9p21 for the associations with carotid IMT and plaque. Multivariate permutation analyses demonstrated that carriers of the T allele of SNP rs1333040 were significantly associated with thicker common carotid artery (CCA) IMT (p = 0.021) and internal carotid artery (ICA) IMT (p = 0.033). The risk G allele of SNP rs2383207 was associated with ICA IMT (p = 0.007). Carriers of the C allele of SNP rs1333049 were found to be significantly associated with thicker ICA IMT (p = 0.010) and the greater risk for the presence of carotid plaque (OR = 1.57 for heterozygous carriers; OR = 1.75 for homozygous carriers). Haplotype analysis showed a global p value of 0.031 for ICA IMT and 0.115 for the presence of carotid plaque. Comparing with the other haplotypes, the risk TGC haplotype yielded an adjusted p value of 0.011 and 0.017 for thicker ICA IMT and the presence of carotid plaque respectively. Further analyzing the data separated by sex, the results were significant only in men but not in women.

**Conclusions:**

Chromosome 9p21 had a significant association with carotid atherosclerosis, especially ICA IMT. Furthermore, such genetic effect was in a gender-specific manner in the Han Chinese population.

## Introduction

A recent discovery has noted that variation of single nucleotide polymorphisms (SNPs) on chromosome 9p21 is highly associated with the risk of coronary artery disease (CAD) and myocardial infarction (MI) [1], [2], [3]. Similarly, this risk region has also been demonstrated to be significantly associated with the atherosclerotic stroke subtype [4]. Given that atherosclerosis is considered as the pathology underlying the majority of CAD and atherosclerotic stroke, it is reasonable to consider that atherosclerosis is one of the mechanisms for 9p21-related events.

Carotid intima-media thickness (IMT) and plaque have been shown to be good subclinical atherosclerotic markers and independent predictors for future cardiovascular events [5], [6]. Despite both phenotypes correlate well to the pathology and clinically defined atherosclerosis, they represent distinct traits [7]. It has been proposed that IMT changes reflect an adaptive process and represent as an early carotid atherosclerosis, while plaque formation may reflect a more significant alteration in vessel wall and represent an estimate of more advanced status [8], [9]. Thus, these two aspects of atherosclerosis measures assay different biological aspects of atherogenesis. Recently, there is evidence that carotid IMT and plaque are largely controlled by genetics with heritability estimates ranged from 30%–60% for IMT [10], [11] and about 28% for carotid plaque [12], [13]. Therefore, both IMT and plaque can be used as independent and compensatory surrogate markers for genetic studies of atherosclerosis.

To date, only few studies have investigated the association of this newly identified risk 9p21 locus with carotid atherosclerosis. In a recent study recruiting subjects from the Atherosclerosis Risk in Communities (ARIC) Study, the sequence variation on chromosome 9p21 was associated with carotid atherosclerosis risk among whites but not in African-American population [14]. In a population-based, prospective, Italian study, the variant alleles on 9p21 were not only found to influence the development but also the progression of carotid atherosclerosis [15]. However, another Finnish Study [16] and British Caucasian population study [17] demonstrated no association between the variant alleles and burden of carotid atherosclerosis. Yet, replication studies in other populations, such as Asians, would be warranted.

Therefore, we conducted a study using a stroke- and MI- free Han Chinese population resided at southern Taiwan to evaluate the risk of chromosome 9p21 for pre-clinical atherogenic phenotypes, including IMT and plaque formation. As sex may contribute to the genetic predisposition [14], [18], sex-specific effect of chromosome 9p21 was also tested.

## Results

Among the 1083 stroke- and MI-free participants, carotid IMT data are available for 1074 subjects, plaque index data for 810 subjects and both sets of data for 801subjects. The clinical, demographic and ultrasonic data are shown in Table 1. The mean and SD of age was 52.16 ± 11.79 years (range from 21–87 years). Men accounted for 45.8% of the total population. Men had significantly thicker carotid IMT in all three segments (p = 3.33×10^−7^ for common carotid artery (CCA); p = 1.83×10^−8^ for bifurcation (Bif); and p = 6.51×10^−17^for internal carotid artery (ICA)) and greater risk for the presence of carotid plaque (p = 1.45×10^−5^) than women (Table 1). The genotype distributions of the four selected SNPs (rs1333040, rs2383207, rs10757278, and rs1333049) did not deviate from Hardy-Weinberg equilibrium (HWE). All four SNPs were in the same linkage disequilibrium (LD) block (Figure S1). Two SNPs (rs10757278 and rs1333049) had high r^2^ of 0.95 and thus only rs1333049 was presented hereafter.

**Table 1:** Characteristics of the study participants.

	All	Men	Women	
	N = 1083	N = 496	N = 587	
	n (%) or mean± SD			p-value (men vs. women)
Age, yr	52.16±11.79	52.31±12.16	52.02±11.48	0.68
Hypertension	287 (26.50)	135 (27.22)	152 (25.89)	0.62
Diabetes	86 (7.97)	50 (10.10)	36 (6.16)	0.02
Hypercholesterolemia	175 (16.23)	80 (16.16)	95 (16.27)	0.96
Body mass index	24.25±3.49	25.09±3.24	23.53±3.55	5.35×10^−13^
Current smoker	106 (10.02)	98 (20.29)	8 (1.39)	2.03×10^−24^
Carotid IMT	n = 1074	n = 490	n = 584	
CCA (mm)	0.61±0.14	0.63±0.16	0.59±0.12	3.33×10^−7^
Bif (mm)	0.64±0.13	0.67±0.14	0.62±0.12	1.83×10^−8^
ICA (mm)	0.50±0.10	0.53±0.11	0.48±0.08	6.51×10^−17^
Carotid plaque	n = 810	N = 323	N = 487	
No plaque	534 (65.9%)	189 (58.5%)	345 (70.8%)	1.45×10^−5^
Any plaque	276 (34.1%)	134 (41.5%)	142 (29.2%)	

IMT: intima-media thickness.

CCA: common carotid artery.

Bif: bifurcation.

ICA: internal carotid artery.

Associations of the three SNPs with carotid IMT and plaque are shown in Table 2 and Table 3 respectively. Only adjusted permutation p values are presented. Carriers of the T allele of SNP rs1333040 had a significantly higher IMT at the segment of CCA IMT (p = 0.021) and ICA IMT (p = 0.033). Carriers of the G allele of SNP rs2383207 had higher ICA IMT (p = 0.007). For the SNP rs1333049, risk C allele was significantly associated with higher ICA IMT (p = 0.010). Of the tested SNPs, only rs1333049 showed significant association with the presence of carotid plaque. Subjects with the GC genotype of rs1333049 had a 1.57-fold (95%CI = 1.06–2.33; p = 0.024) risk for the presence of carotid plaque, compared with those with the GG genotype. Similarly, the CC genotype frequency in the carotid plaque group was higher (adjusted OR = 1.75, 95%CI = 1.12–2.75; p = 0.015) than GG genotype.

**Table 2:** Association of chromosome 9p21 SNPs with carotid intima-media thickness (IMT) (n = 1074).

	IMT, Mean±SD (mm)			p* value
rs1333040	CC (n = 99)	CT (n = 436)	TT (n = 516)	
CCA IMT	0.60±0.11	0.60±0.13	0.62±0.15	**0.021**
Bif IMT	0.63±0.11	0.64±0.12	0.65±0.14	0.125
ICA IMT	0.50±0.08	0.49±0.09	0.51±0.11	**0.033**

rs2383207	AA (n = 136)	AG (n = 460)	GG (n = 454)	
CCA IMT	0.61±0.12	0.60±0.14	0.61±0.15	0.192
Bif IMT	0.64±0.11	0.64±0.12	0.65±0.14	0.117
ICA IMT	0.49±0.08	0.49±0.09	0.51±0.11	**0.007**

rs1333049	GG (n = 311)	GC (n = 493)	CC (n = 255)	
CCA IMT	0.61±0.13	0.61±0.14	0.61±0.15	0.592
Bif IMT	0.64±0.11	0.64±0.14	0.65±0.14	0.536
ICA IMT	0.49±0.09	0.50±0.09	0.51±0.12	**0.010**

*Permutation p value after being adjusted for age, sex, hypertension, diabetes, hypercholesterolemia, body mass index and current smoking.

CCA: common carotid artery.

Bif: bifurcation.

ICA: internal carotid artery.

**Table 3:** Genotype distribution of chromosome 9p21 SNPs according to carotid plaque status (n = 810).

SNP/genotype	Carotid plaque, N (%)		OR (95%CI)	p* value
	No plaque	Any plaque		
rs1333040				
CC	48 (9.21)	22 (8.03)	1.00	
CT	225 (43.19)	103 (37.59)	1.06 (0.58–1.93)	0.849
TT	248 (47.60)	149 (54.38)	1.39 (0.78–2.55)	0.274
rs2383207				
AA	72 (13.87)	29 (10.66)	1.00	
AG	222 (42.77)	118 (43.38)	1.40 (0.84–2.39)	0.179
GG	225 (43.35)	125 (45.96)	1.56 (0.93–2.67)	0.071
rs1333049				
GG	166 (31.62)	65 (23.72)	1.00	
GC	238 (45.33)	136 (49.64)	1.57 (1.06–2.33)	**0.024**
CC	121 (23.05)	73 (26.64)	1.75 (1.12–2.75)	**0.015**

*Permutation p value after being adjusted for age, sex, hypertension, diabetes, hypercholesterolemia, body mass index and current smoking

To examine the sex-specific effect, we further elucidated the above significant associations of candidate SNPs and atherosclerosis phenotypes by sex stratification. Sex-specific analysis showed that the significant association only existed in men and no significant associations were observed in women (Table 4). Men carrying the risk T allele of SNP rs1333040 had thicker IMT at the CCA (p = 0.015) and ICA (p = 0.012) segments. The G allele of rs2383207 was associated with increased ICA IMT (p = 0.004). Similarly, the C allele of rs1333049 was also shown to be related with ICA IMT (p = 0.002) and the presence of carotid plaque. Among the male subjects, the OR (95% CI) for the presence of carotid plaque were 1.87 (1.01–3.56) for the GC genotype and 2.45 (1.22–5.01) for the CC genotype compared with the GG genotype. In addition to stratifying the data by sex, the above significant associations was re-examined in a regression model to exam the interaction between sex and genotypes. There was a borderline interaction effect between sex and rs1333040 for CCA IMT (p = 0.060); while significant interaction effects between sex and rs2383207 (p = 0.044), sex and rs1333049 (p = 0.016) for ICA IMT. However, for the carotid plaque, the number of cases in the presence of carotid plaque group was relatively small (for example, only 134 men had plaque) and the interaction difference for rs1333049 did not reach statistical significance (p = 0.540). Further, the interaction by sex might be potentially confounded by smoking for the most current smokers are males in this study. However, there was no statistical difference between smoking and genetic variation for carotid atherosclerosis after stratified analysis by smoking (data not shown).

**Table 4:** Stratification analysis for association of chromosome 9p21 SNPs with carotid intima-media thickness (IMT)/plaque by sex.

	Men		Women	
	β value	p* value	β value	p* value
CCA IMT‡	n = 490		n = 584	
rs1333040 (T)†	0.022	**0.015**	0.004	0.519
ICA IMT‡				
rs1333040 (T)†	0.019	**0.012**	0.002	0.642
rs2383207 (G)†	0.021	**0.004**	0.003	0.487
rs1333049 (C)†	0.022	**0.002**	0.001	0.815

Any vs. no plaque	n = 134 vs. 189		n = 142 vs. 345	
	OR (95%CI)	p* value	OR (95%CI)	p* value
rs1333049				
GG	1.00		1.00	
GC	1.87 (1.01–3.56)	**0.051**	1.36 (0.83–2.27)	0.228
CC	2.45 (1.22–5.01)	**0.013**	1.36 (0.75–2.47)	0.318

*permutation p value after being adjusted for age, hypertension, diabetes, hypercholesterolemia, body mass index and current smoking.

‡ The IMT was measured as a continuous variable.

† Parentheses represented the risk allele of candidate SNPs.

CCA: common carotid artery; ICA: internal carotid artery.

The association of haplotypes comprising these three SNPs (rs1333040, rs2383207 and rs1333049) was further investigated. The global p values were 0.031 for ICA IMT and 0.115 for the presence of plaque. As compared with other haplotypes, the risk TGC haplotype had a higher risk for ICA IMT and the presence of carotid plaque with an adjusted p value of 0.011 and 0.017 respectively in overall study subjects. The data were then re-analyzed by sex stratification. For men, the global p values were 0.028 for ICA IMT and 0.028 for the presence of plaque. The risk TGC haplotype was significantly associated with ICA IMT (adjusted p = 0.007, beta coefficient = 0.01) and the presence of carotid plaque (adjusted p = 0.012, OR = 1.57) in males. However, no individual haplotype was significantly associated with carotid IMT or plaque in female subjects.

## Discussion

In this study, we investigated the relationship between SNPs at chromosome 9p21 and carotid atherosclerosis measured as carotid IMT and plaque. We found that (1) SNPs at this region were associated with both atherosclerotic traits; (2) the genetic effect of 9p21 was only found in men but not in women, and (3) the genetic association was found on IMT at both CCA and ICA segments, especially at ICA. Given that atherosclerosis is a common pathogenesis underlying the majority of CAD and stroke, our findings provide additional insight to understand the mechanistic basis of 9p21 on these clinical consequences.

Different atherosclerosis phenotypes have been used for evaluating the association of 9p21 in the carotid artery, including IMT [14], [15], [16], [17], stenosis [15], and plaque [15]. The previous relevant studies and ours were summarized in Table 5. The Finnish study included two population-based cohorts of different age groups (2277 individuals between 24–39 years old and 1295 individuals between 46–76), and found no evidence of an association between rs1333049 and CCA IMT in either cohort [16]. Another study enrolling 1425 members of 248 Caucasian families ascertained through a hypertensive proband also failed to provide the evidence of association between four 9p21SNPs (rs1333049, rs7044859, rs496892 and rs7865618) and CCA IMT [17]. In contrast, a data analysis of the ARIC Study revealed an association between SNP rs10757274 (r^2^ =  0.91 with rs1333049 in the HapMap [19] Chinese samples) and carotid atherosclerosis in the 11085 white subjects but not in the 4018 African-American participants [14]. However, the definition of carotid atherosclerosis in this study was mixed phenotypes, as high IMT (mean of CCA, ICA and bifurcatin IMT >1.0 mm) or the presence of carotid plaque. With mean IMT (i.e. combined CCA, ICA and bifurcation IMT), no significant association was found in this study [14]. Our study is the first to report a significant relationship of 9p21 on carotid atherosclerosis in Asians. The genetic variation in our study is associated with a risk for increased CCA, ICA IMT and carotid plaque. Leading explanations for these discrepant findings include the presence of undetected population stratification at 9p21 as well as differences of races, environment and the method to assess the carotid atherosclerosis, especially on the segment of IMT measurement. Population stratification is unlikely as an explanation for our case-control association result since we have tested for it in part of our cohort and no detectable population stratification was found [20].

**Table 5:** Relevant studies of polymorphisms at 9p21 and carotid atherosclerosis.

Study population	Study design, sample size, Age (range or mean±SD)	Study SNPs	Phenotypes	Findings
Italian [15]	Prospective*, n = 769, age: 40–79	rs1333049, rs10757274	1. Atherosclerosis progression§;	1. Significant results in all phenotypes except for CCA IMT;
			2. Incident stenosis>40%;	2. No gender specific effect
			3. Atherosclerosis score†;	
			4. CCA IMT	
Finnish [16]	Two cohorts: Young Finns Study (n = 2277, age: 24–39); Health 2000 cohort (n = 1295, age: 46–76)	rs1333049	CCA IMT	No association in either cohort
British [17]	Family members of hypertensive probands, n = 1425, mean age:49.5± 15.5	rs1333049, rs7044859, rs496892, rs7865618	CCA IMT	No association
White and African-Americans [14]	African-American (n = 4018); White American (n = 11085); overall age: 45–64	rs10757274, rs2383206	1. Mean IMT (i.e. combined CCA, Bif and ICA IMT);	1. No associations for mean IMT;
			2. Carotid atherosclerosis‡	2. Significant association for carotid atherosclerosis among white but not in African-Americans
Chinese (the present study)	healthy volunteers, n = 1083, age: 21–87	rs1333040, rs2383207, rs1333049, rs10757278	1. Different segment IMT over CCA, Bif, ICA;	1. Significant association for CCA and ICA IMT, more prominent at ICA segment;
			2. Carotid plaque	2. Significant association for carotid plaque;
				3. Gender specific effect: only in men, not in women

IMT: intima-media thickness, CCA: common carotid artery, Bif: bifurcation, ICA: internal carotid artery.

*Phenotypes follow-up in the 1990, 1995 and 2000.

§ Atherosclerosis progression: defined by the occurrence of atherosclerotic lesions in segments previously free of atherosclerosis or relative increase in the plaque diameter.

† Atherosclerosis score: calculated by summing the diameter of plaques at 8 locations, including bilateral proximal and distal segments of CCA and ICA.

‡ carotid atherosclerosis: high IMT (mean of CCA, ICA and bifurcatin IMT >1.0 mm) or the presence of carotid plaque.

It is suggested that each carotid artery segment could be influenced by different sets of genes [21] and be prone to variant pathophysiologic mechanisms [22]. Moreover, atherosclerosis at different carotid artery segment was demonstrated to have variant prediction of clinical cardiovascular events [5]. This present study was assessed the relationship between chromosome 9p21 genetic variants and each carotid segment IMT. The results indicated that 9p21 was associated with IMT at both segments of CCA and ICA, and this relationship is particularly pronounced at ICA segment. The ICA IMT was thought to be a better predictor of MI then CCA IMT or the combined CCA, ICA measure [5]. This finding provided more understanding the mechanism of 9p21 in CAD risk.

Atherosclerosis can be viewed as a gradual process from thickening of IMT to plaque formation. In contrast with carotid IMT, the relation between carotid plaque and chromosome 9p21 was seldom studied except for one recent report [15]. Consistent with our findings, that study showed the association of the rs1333049 SNP with the severity of atherosclerosis score (calculated by summing the maximum diameter of CCA, ICA plaques) [15]. This present study successfully demonstrated a relationship between 9p21 and both carotid atherosclerotic phenotypes, indicating the contribution of the risk gene variants in both early and late stages of atherogenesis. This data, along with prior studies demonstrated the associations between this risk genetic region and progression of atherosclerosis [15], abdominal aortic and cerebral aneurysms [23], [24], implicate that the mechanism of this genetic effect on the vascular wall is more complex and could influence various vascular pathogenesis.

A sex-differential relationship seen in this study is similar to the association between heart failure and SNP rs10757274 at 9p21 [14] and that between SNP rs10757274 and abdominal aorta stiffness [18], both in men only. A possible biological explanation is that sex hormones may regulate the expression of these candidate genes [25]. To test if the gene-estrogen interaction is involved, female subjects were stratified by the menopausal status for further analysis. The absence of significant difference between carotid atherosclerosis and the three SNPs at 9p21 in either strata (Tables S1–S2) indicated the lack of association in women was not related to the menopausal status. Since the chromosome 9p21 region has been related to increased risk for MI in both men and women, the genetic effect on women may demonstrate in other intermediate phenotypes rather than carotid IMT or plaque. Further studies are warranted to elucidate the molecular mechanism of 9p21 in women.

There are strengths and limitations in the present study. The chromosome 9p21 association studies have mainly been done in the Caucasian populations and our study is the first to show its relationship in the Asian population and also the sex-specific effect. Our IMT was measured in the plaque free region, therefore, the genetic association for plaque and IMT would not be confounded by the measurement issue. Since our recruited participants were free of MI and stroke, the interpretation of these results can be expanded to the general population although it may not be applicable to high risk populations such as diabetic subjects.

In conclusion, our work suggests that the chromosome 9p21 had a significant association with carotid atherosclerosis, especially ICA IMT. Among the Han Chinese population, the genetic effect was primary found in men but not in women.

## Methods

### Ethics Statement

This study was approved by the Institutional Review Board of Kaohsiung Medical University Hospital. All participants provided written informed consent.

### Study Subjects

Subjects were recruited between January 2006 and December 2008 with an advertisement solicitation at the Kaohsiung Medical University Hospital. After excluding volunteers with a history of stroke (n = 29) and MI (n = 10), a total of 1083 stroke- and MI- free participants were included.

Each participant filled a self-administered questionnaire which includes sociodemographic information, medical history and medication data. Total cholesterol, triglycerides, and glucose levels were measured from venous blood after fasting for at least 8 hours. Hypertension was defined as systolic or diastolic blood pressure ≥140/90 mm Hg, or under anti-hypertensive medications. Diabetes was defined as fasting blood glucose ≥126 mg/dl or known treatment for diabetes. Hypercholesterolemia was defined as serum levels of total cholesterol ≥240 mg/dl.

### Carotid ultrasongraphy studies

#### IMT measurement

Carotid IMT was assessed using Philips HD 11 ultrasonography system equipped with a broadband linear array 3∼12-MHz transducer. All ultrasound measurements were conducted by a experienced technologist who was blind to the patients' clinical data. The far wall of carotid IMT was visualized bilaterally and IMT was measured at plaque-free area of CCA (10–20 mm proximal to the tip of the flow divider), Bif (tip of the flow divider and extending 10 mm proximally) and ICA (proximal 10 mm above the bulb) separately. The carotid IMT was analyzed by the off-line automatic computerized analyzing system (Philips Qlab quantification software). Mean IMT values of both right and left arteries from each segment were taken for analyses. The intra-reader absolute IMT difference and standard deviation (SD) between two readings was 0.01±0.01 mm in CCA, 0.03±0.04 mm in Bif and 0.02±0.01 mm in ICA.

#### Carotid plaque measurement

Carotid plaque, defined as an area of focal protrusion into the lumen at least 50% greater than the surrounding wall thickness, was assessed by the same equipment used in carotid IMT measurement. Presence of carotid plaques at 10 locations in the carotid arteries (proximal and distal CCA, Bif, ICA and external carotid artery) was recorded. The carotid plaque reading protocol was performed offline and all the ultrasonic data were read by a single neurologist (HFL).

### Genotyping

Genomic DNA was isolated from whole blood using a commercial available kit (Puregene from Gentra, Research Triangle, NC). Four SNPs (rs10757278, rs2383207, rs1333040 and rs1333049) at chromosome 9p21 were selected on the basis of previous studies consistently reporting as key SNPs to CAD and stroke [1], [3], [26]. Genotypes were determined using TaqMan technology (Applied Biosystems, Foster City, California). The genotyping success rates of the four SNPs ranged from 96.3% to 98.7%.

### Statistical analysis

Allele frequencies at each SNP were estimated by direct genotypes counting. HWE was tested for the relationship between allele and genotype frequencies by using the *x* ^2^ test. HWE states both allele and genotype frequencies in a population remain constant. Deviated from the HWE can cause false positive of observed association in the data. The LD, which is a phenomenon whereby genetic variants are associated, between the tested SNPs was calculated by the Haploview software (version 4.2) [27]. We assumed an additive model in the present analyses. The associations of genotypes with carotid IMT were analyzed by multivariate regression models with adjustment for conventional risk factors (age, sex, diabetes, hypertension, current smoking, body mass index and hypercholesterolemia). For carotid plaque, logistic regression analysis was used to evaluate the difference of genotype distribution between with and without plaque group. To assess the sex-specific effect, we analyzed male and female data separately and further confirmed in the regression model by adding an interaction variable (genotypes*sex) with adjustment for conventional risk factors.

The package Hap-Clustering of R software (version 2.9.0) [28] was used to test for the haplotype-phenotype association. The haloptype global p value was obtained by evaluating the frequencies of overall possible haplotypes between cases and controls; whereas the haplotype-specific p value was for a particular haplotype between cases and controls. Multiple testing corrections were performed using the max (T) permutation procedure for 10,000 times to obtain empirical p value by the PLINK software [29]. A two-tailed P value less than 0.05 was considered statistically significant. Statistical analyses were performed with the SPSS 12.0 software (SPSS Inc., Chicago, Illinois, USA).

## Supporting Information

Figure S1 **Analysis of linkage disequilibrium (LD).**
(DOC)

Table S1 **Chromosome 9p21 SNPs with carotid IMT/plaque in women without menopause.**
(DOC)

Table S2 **Chromosome 9p21 SNPs with carotid IMT/plaque in women with menopause.**
(DOC)
