# Supplementary material for: Sex Differential Genetic Effect of Chromosome 9p21 on Subclinical Atherosclerosis
Source: PLoS One. 2010 Nov 30;5(11):e15124. doi: 10.1371/journal.pone.0015124 (PMC2994883; doi:10.1371/journal.pone.0015124)

Figure S1.

Analysis of linkage disequilibrium (LD). LD pattern between four SNPs, rs1333040, rs2383207, rs10757278 and rs1333049 were derived from the genotyping data from the present study subjects. The pairwise correlation between the SNPs was measured as D’ (A) and r^2^ (B).

(A)


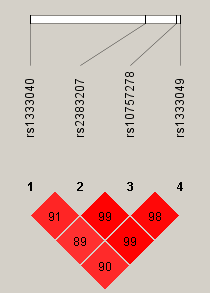


(B)


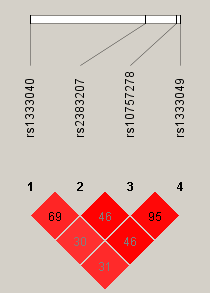

Supplement: Figure S1 — Analysis of linkage disequilibrium (LD). (DOC) [file pone.0015124.s001.doc]
