# Supplementary material for: Sex Differential Genetic Effect of Chromosome 9p21 on Subclinical Atherosclerosis
Source: PLoS One. 2010 Nov 30;5(11):e15124. doi: 10.1371/journal.pone.0015124 (PMC2994883; doi:10.1371/journal.pone.0015124)
Supplement: Table S1 — Chromosome 9p21 SNPs with carotid IMT/plaque in women without menopause. (DOC) [file pone.0015124.s002.doc]

Table S1. Chromosome 9p21 SNPs with carotid IMT/plaque in women without menopause†

| SNPs | phenotype | Genotype | | | p-value* |
| --- | --- | --- | --- | --- | --- |
| rs1333040 | IMT, mean±SD | TT (n=107) | CT (n=100) | CC (n=22) |  |
|  | CCA | 0.52±0.09 | 0.51±0.08 | 0.54±0.07 | 0.554 |
|  | Bif | 0.57±0.11 | 0.56±0.10 | 0.59±0.10 | 0.923 |
|  | ICA | 0.46±0.08 | 0.44±0.07 | 0.49±0.09 | 0.988 |
|  | Carotid plaque, n(%) |  |  |  |  |
|  | No plaque | 58 (46.03) | 58 (46.03) | 10 (7.41) |  |
|  | Any plaque | 17 (56.67) | 9 (30.00) | 4 (13.33) | 0.770 |
| rs2383207 | IMT, mean±SD | GG (n=105) | AG (n=98) | AA (n=28) |  |
|  | CCA | 0.53±0.09 | 0.50±0.08 | 0.55±0.08 | 0.704 |
|  | Bif | 0.58±0.11 | 0.55±0.10 | 0.60±0.09 | 0.769 |
|  | ICA | 0.46±0.08 | 0.44±0.07 | 0.48±0.08 | 0.607 |
|  | Carotid plaque n(%) |  |  |  |  |
|  | No plaque | 54(42.19) | 59(46.09) | 15(11.72) |  |
|  | Any plaque | 19(63.33) | 7(23.33) | 4(13.33) | 0.235 |
| rs1333049 | IMT, mean±SD | GG (n=71) | CG (n=101) | CC (n=59) |  |
|  | CCA | 0.53±0.08 | 0.51±0.08 | 0.52±0.09 | 0.324 |
|  | Bif | 0.58±0.09 | 0.56±0.11 | 0.57±0.10 | 0.619 |
|  | ICA | 0.46±0.07 | 0.44±0.08 | 0.47±0.08 | 0.437 |
|  | Carotid plaque n(%) |  |  |  |  |
|  | No plaque | 41 (32.03) | 59 (46.09) | 28 (21.88) |  |
|  | Any plaque | 7 (23.33) | 12 (40.00) | 11 (36.67) | 0.149 |

*p value after being adjusted for age, hypertension, diabetes, hypercholesterolemia and current smoking

†Menopause was defined by women>=55 years or self-reported with post-menopause status
