# Supplementary material for: Sex Differential Genetic Effect of Chromosome 9p21 on Subclinical Atherosclerosis
Source: PLoS One. 2010 Nov 30;5(11):e15124. doi: 10.1371/journal.pone.0015124 (PMC2994883; doi:10.1371/journal.pone.0015124)
Supplement: Table S2 — Chromosome 9p21 SNPs with carotid IMT/plaque in women with menopause. (DOC) [file pone.0015124.s003.doc]

Table S2. Chromosome 9p21 SNPs with carotid IMT/plaque in women with menopause†

| SNPs | phenotype | Genotype | | | p-value* |
| --- | --- | --- | --- | --- | --- |
| rs1333040 | IMT, mean±SD | TT (n=165) | CT (n=149) | CC (n=30) |  |
|  | CCA | 0.63±0.13 | 0.63±0.12 | 0.65±0.12 | 0.973 |
|  | Bif | 0.66±0.12 | 0.66±0.11 | 0.63±0.09 | 0.417 |
|  | ICA | 0.49±0.08 | 0.49±0.08 | 0.49±0.08 | 0.442 |
|  | Carotid plaque, n(%) |  |  |  |  |
|  | No plaque | 102 (48.11) | 91 (42.92) | 19 (8.96) |  |
|  | Any plaque | 56(50.45) | 46 (41.44) | 9 (8.11) | 0.631 |
| rs2383207 | IMT, mean±SD | GG (n=142) | AG (n=152) | AA (n=46) |  |
|  | CCA | 0.62±0.13 | 0.64±0.12 | 0.65±0.12 | 0.569 |
|  | Bif | 0.66±0.11 | 0.66±0.12 | 0.64±0.10 | 0.221 |
|  | ICA | 0.49±0.08 | 0.49±0.08 | 0.49±0.08 | 0.672 |
|  | Carotid plaque, n(%) |  |  |  |  |
|  | No plaque | 93 (44.93) | 81 (39.61) | 32 (15.46) |  |
|  | Any plaque | 41 (36.94) | 58 (52.25) | 12 (10.81) | 0.883 |
| rs1333049 | IMT, mean±SD | GG (n=103) | CG (n=164) | CC (n=78) |  |
|  | CCA | 0.64±0.11 | 0.63±0.13 | 0.62±0.13 | 0.478 |
|  | Bif | 0.66±0.10 | 0.66±0.12 | 0.65±0.12 | 0.503 |
|  | ICA | 0.49±0.07 | 0.49±0.08 | 0.49±0.08 | 0.897 |
|  | Carotid plaque, n(%) |  |  |  |  |
|  | No plaque | 67 (31.60) | 94 (44.34) | 51 (24.06) |  |
|  | Any plaque | 30 (27.03) | 59 (53.15) | 22 (19.82) | 0.984 |

*p value after being adjusted for age, hypertension, diabetes, hypercholesterolemia and current smoking

†Menopause was defined by women>=55 years or self-reported with post-menopause status
